# Invasive fungal pathogens

**DOI:** 10.2471/BLT.23.020323

**Published:** 2023-03-01

## Abstract

Increased focus on invasive fungal pathogens promises to drive improvements in reporting, diagnostics and treatments. Tatum Anderson reports.

Professor Arnaldo Lopes Colombo sees a range of critically ill patients in the intensive care units he visits in São Paulo, Brazil. What they all have in common is protracted exposure to invasive medical procedures and a failure to respond to treatment that should normally be helping. 

“Typically, they will have been treated with several antibiotics and then gotten worse,” Colombo says. “That’s when I am called in.”

At the Federal University of São Paulo, Brazil, Colombo specializes in identifying fungal pathogens and offering guidance regarding the diagnosis and management of the diseases they cause. 

“When I see patients being treated for tuberculosis for a second time without laboratory confirmation of infection, I ask the clinicians if they have looked for *Aspergillus*,” he says. “Most of them have never heard of it.” 

Like many fungal pathogens, *Aspergillus *is found almost everywhere, and people typically inhale thousands of its spores every day. For healthy individuals this is not a problem, but for those whose immune system has been weakened or people with pre-existing chronic pulmonary conditions it can lead to aspergillosis, a disease that can give rise to chronic lung dysfunction.

As Colombo knows well, chronic pulmonary aspergillosis can easily be mistaken for tuberculosis. “Misdiagnosis often leads to inappropriate treatment, which then results in further deterioration of the patient which can include organ failure and death.” he says. 

*Aspergillus *is one of 200 fungi known to cause disease in humans, the more pernicious of which cause invasive infections. These are defined as infections other than those affecting the skin or mucosal tissue, and include infections of the bloodstream which can impact the immune system or otherwise cause serious systemic harm. 

The burden of disease imposed by invasive fungi has yet to be established because of the lack of data, but emerging evidence suggests that the incidence and geographic range of fungal diseases are considerable and may be expanding.

One reason for the lack of evidence, is the lack of awareness regarding fungal infections on the part of clinicians. “The index of suspicion for fungal infections is low to non-existent,” explains Dr Rita Oladele, a clinician and head of the Department of Medical Microbiology at the University of Lagos, Nigeria, who like Colombo is often called upon to solve puzzling cases. “For example, doctors treating patients with HIV who present with severe and persistent headaches rarely consider the possibility of cryptococcal meningitis, despite its prevalence.”

Caused by the fungus *Cryptococcus neoformans, *cryptococcal meningitis is estimated to account for up to 15% of HIV-related deaths globally and as much as 50% in resource-limited settings. Using advocacy based on painstakingly accumulated epidemiological data, Oladele is raising awareness of the disease in her country, but she believes that there is still a long way to go (see interview in this issue).

“The number of invasive fungal infections is only going to increase.”

John Perfect

For doctors already alert to the threat posed by fungal pathogens, a lack of diagnostic tools stands in the way of effective diagnosis and reporting. Point-of-care tests for invasive fungal infections do exist, but they tend to be expensive, leaving doctors reliant on laboratory confirmation, which takes longer and is not always available.

Despite the obstacles faced, invasive fungal pathogens are now becoming more visible. This is in part because of headline-grabbing outbreaks of some of the more distressing diseases they cause, reported as co-morbidities with coronavirus disease 2019 (COVID-19).

These include outbreaks of mucormycosis in India. Caused by a group of moulds called mucormycetes, mucormycosis can result in distinctive black lesions on the bridge of the nose or inside of the mouth and, in some settings, mortality rates as high as 50%. 

According to Professor Arunaloke Chakrabarti, who until recently lead the Mycology Division in the Department of Medical Microbiology at the Postgraduate Institute of Medical Education and Research in Chandigarh, India, close to 50 000 cases of mucormycosis were reported in India in 2021, the majority of those infected being people with (or recently recovering from) COVID-19, immunosuppressed individuals, or individuals with underlying conditions such as diabetes. 

Many of the infections occurred in hospitals. Trying to understand why that was, Chakrabarti carried out multiple studies including one to establish where mucormycetes spores were getting in. “We found that they were colonizing ducts of central air conditioning systems around hospitals and, due to the absence of HEPA (high-efficiency particulate absorbing) filters at the end of the ducts, spores were able to get through,” he says.

*Candida auris* is another fungal pathogen to have made its presence felt in hospitals, attracting particular attention because, in certain cases, it has shown resistance to all four commonly used antifungal drugs. 

The pathogen was recognized in South Korea in the late 1990s and came to prominence after a case was reported in Japan in 2009. It has since spread around the world, often in hospitals where it gives rise to bloodstream, wound and ear infections. 

“*Candida auris* is just everywhere and it’s resistant to some hospital-grade disinfectants,” says Professor Nelesh Govender of South Africa’s National Institute for Communicable Diseases. “So once it gets into a hospital environment you just can't get rid of it.” 

While it is perhaps understandable that emergent ‘super-fungi’ are grabbing headlines, it is the suspected increase in cases driven by an ever-expanding at-risk population that is holding the attention of most medical mycologists. 

“Until we establish credible baselines it is going to be difficult to identify clear trends, but as at-risk subgroups continue to expand, notably those people developing and being treated for noncommunicable diseases such as diabetes and cancers and those undergoing invasive surgical procedures, it seems likely that the number of invasive fungal infections is only going to increase,” says Professor John Perfect, Director of the Duke University Mycology Research Unit at Duke University in the United States of America.

Ironically, fungal infections appear to be one of the prices paid for health-care systems that are getting better at treating the chronically sick and the acutely ill. Chemotherapy and immunotherapy for cancer, and solid organ transplantations save more lives than ever today, but the immune system suppression they require present opportunities for pathogenic fungi to become established. The same is true of treatments for chronic obstructive pulmonary disorder, tuberculosis, HIV and diabetes mellitus.

“Misdiagnosis often leads to inappropriate treatment.”

Arnaldo Lopes Colombo

Another possible driver of upward trends is the widening geographical distribution of fungal infections possibly resulting from climate change. “More research is needed, but my feeling is that this is already happening,” says Chakrabarti, who points out that fungi flourish at 20-25 degrees centigrade, and thus struggle inside the human body. “What happens if because of global warming, fungi adapt to higher temperatures?” Chakrabarti asks.

It was in response to these developing challenges that in October 2022, the World Health Organization (WHO) published the first-ever fungal priority pathogens list (WHO FPPL). Divided into three categories (critical, high and medium priority), the list constitutes the first global effort to systematically prioritize fungal pathogens, reflecting unmet research and development needs and assessed public health importance. 

“We’re preparing for the future as the evidence comes in,” says Dr Haileyesus Getahun, director of the WHO’s Department of Global Coordination and Partnership on Antimicrobial Resistance, and one of the list’s architects. “The hope is that the list will stimulate more focused research on the disease burden imposed by fungal pathogens, as well as their distribution, ecology, and links to global warming. The ultimate aim is to inform policy development and better prepare the managers and clinicians on the front line.” 

The next step, Getahun explains, is to assess the current state of play with regard to the research and development pipeline of treatments and diagnoses relevant to the pathogens on the list. “Based on the pipeline analysis we can consult with industry, scientific groups and researchers and pinpoint the gaps and the deficiencies,” Getahun says. “Additionally, the analysis will serve to inform donors where they can best invest resources, bearing in mind the other challenges we face, notably in regard to bacterial drug resistance which kills around 1.3 million people every year directly, and contributes to the deaths of another 5 million.” 

The list is also designed to encourage innovation, for example by focusing research and development on new antifungal agents that can get round the growing problem of resistance by employing new mechanisms.

Perfect is hoping that the list will bring fungal pathogens out of the shadows, noting that is has already focused attention on mycology in a way that was not previously the case. Chakrabarti is counting on the list inspiring researchers in low- and middle-income countries to focus on the pathogens that are of particular concern for them.

Oladele hopes the list will also encourage work on diagnostics. Thanks to the evidence-based advocacy of Oladele and her colleagues, clinicians in Nigeria screen HIV patients with advanced disease for *Cryptococcus*, making it possible to catch infections and start antifungal treatment early. She’d like to see similar tests for other pathogens. “This would have a massive knock-on effect for surveillance, treatment guidelines, public policy, stewardship and perhaps most importantly, awareness among clinicians,” she says. “That would be a very welcome development.”

**Figure:**
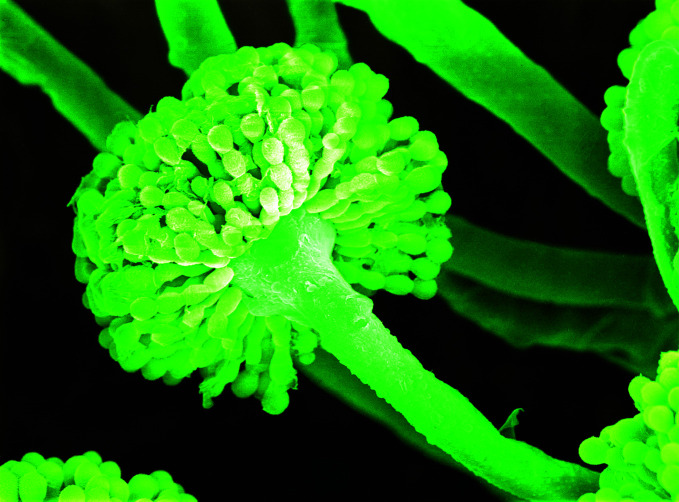
Scanning electron micrograph of *Aspergillus* mould producing spores, computer-coloured green.

Wellcome Collection/David Gregory & Debbie Marshall

**Figure:**
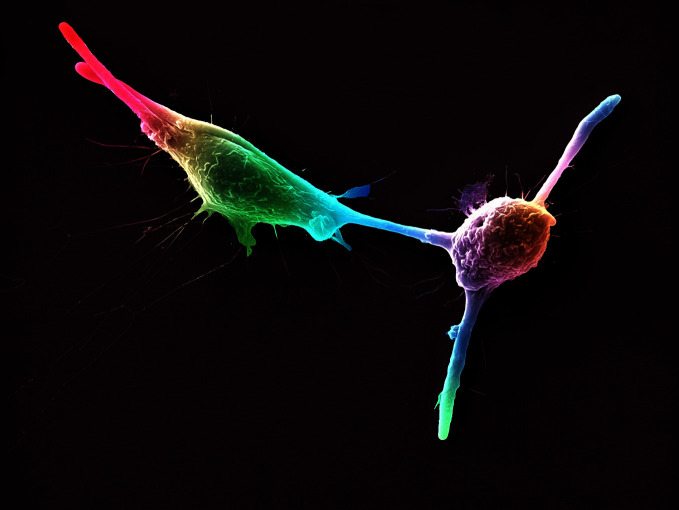
False-coloured scanning electron micrograph of two macrophage cells infected with *Candida* yeast spores.

Wellcome Collection/Kevin Mackenzie

